# The Design of SimpleITK

**DOI:** 10.3389/fninf.2013.00045

**Published:** 2013-12-30

**Authors:** Bradley C. Lowekamp, David T. Chen, Luis Ibáñez, Daniel Blezek

**Affiliations:** ^1^National Library of Medicine, Office of High Performance Computing and Communications, National Institutes of HealthBethesda, MD, USA; ^2^Medical Science and ComputingRockville, MD, USA; ^3^Kitware Inc.Clifton Park, NY, USA; ^4^Biomedical Engineering Department, Mayo Graduate School of MedicineRochester, MN, USA

**Keywords:** software design, Insight Toolkit, segmentation, software development, image processing software, image processing and analysis

## Abstract

SimpleITK is a new interface to the Insight Segmentation and Registration Toolkit (ITK) designed to facilitate rapid prototyping, education and scientific activities via high level programming languages. ITK is a templated C++ library of image processing algorithms and frameworks for biomedical and other applications, and it was designed to be generic, flexible and extensible. Initially, ITK provided a direct wrapping interface to languages such as Python and Tcl through the WrapITK system. Unlike WrapITK, which exposed ITK's complex templated interface, SimpleITK was designed to provide an easy to use and simplified interface to ITK's algorithms. It includes procedural methods, hides ITK's demand driven pipeline, and provides a template-less layer. Also SimpleITK provides practical conveniences such as binary distribution packages and overloaded operators. Our user-friendly design goals dictated a departure from the direct interface wrapping approach of WrapITK, toward a new facade class structure that only exposes the required functionality, hiding ITK's extensive template use. Internally SimpleITK utilizes a manual description of each filter with code-generation and advanced C++ meta-programming to provide the higher-level interface, bringing the capabilities of ITK to a wider audience. SimpleITK is licensed as open source software library under the Apache License Version 2.0 and more information about downloading it can be found at http://www.simpleitk.org.

## 1. Introduction

The proper practice of the scientific method requires the systematic verification of reproducibility of published reports (Popper, 1934). Ideally, this verification should be performed by independent observers for it to be trustable (Popper, 1968). In the context of computational science, the reports of scientific research must include the means and the complete details required to enable independent groups to fully replicate the published results. In particular, they should include: data, reports, list of experimental parameters, and software.

Open source software provides public implementations of state of the art algorithms, that facilitate the accelerated advancement of a field, through a more efficient research lifecycle and more practical education. The National Library of Medicine's Insight Segmentation and Registration Toolkit (ITK) is a leading open source software library for biomedical image analysis. Since 1999, it has been used in fields as diverse as brain registration for neuroscience, microscopy image analysis, radiation treatment planning, image segmentation for brain tumors, and processing of electron microscopy, as well as non-medical applications such as satellite imagery and industrial inspection.

Our fundamental goal in developing SimpleITK was to grow the user community of ITK. Whereas direct use of the ITK programming interface requires expertise in templated C++, SimpleITK was designed to be accessible from a variety of higher level languages. Furthermore SimpleITK has a straightforward interface that requires no knowledge of the intricacies of ITK's templated types. By lowering the bar to access ITK's portfolio of image processing algorithms we hope to reach the domain scientist and to further the goals of open source and open science.

### 1.1. The insight segmentation and registration toolkit

The Insight Segmentation and Registration Toolkit (ITK) was originally conceived as open software tools for the analysis of the Visible Human Project by the National Library of Medicine (NLM) with partnership from six other institutes at the National Institutes of Health (NIH). During the initial development the mission of ITK was outlined as: a software foundation for future research, an archival repository of image processing algorithms, a catalog of validation techniques, as well as a platform for advanced product development (Yoo et al., 2002). NLM has continued to support ITK through Algorithm Adaptors and Data Distribution (A2D2) programs and on going maintenance, while trying to foster the development of a sustainable open source community. In 2010 NLM initiated a major revision and refactoring of the toolkit funded by the American Recovery and Reinvestment Act. Among the objectives outlined is to simplify ITK.

Through the contributions of the ITK community and continued funding the scope of ITK has continued to grow. The version 4 refactoring separated ITK into a modular structure which now contains over 100 modules. The segmentation algorithms available in ITK include region growing, level sets, Markov random field classifiers, watersheds and other statistical classifiers. The registration framework is designed to be modular with the distinct parts for a transform, interpolator, transform, optimizer, and image similarity metric along with support for multi-resolution methods. ITK also contains data-structures for spatial object, histograms, finite element meshes, quad-edge meshes, neural networks, images and narrow band level-sets. There are a large number of third party libraries that are supported and used for input and output. Additionally there are numerous image filters algorithms available including mathematical morphology, smoothing, deconvolution, distance maps, fast marching, image fusion, image statistics, geometric filters, and image sources among many others. ITK contains a wealth of algorithms, interfaces and data-structures to provide a platform for research in algorithm development and a collection of image processing algorithms.

The design of ITK focuses on providing a powerful and flexible platform to allow for research, experimentation and development of algorithms. To that end one of the notable implementation details in ITK is the extensive use of C++ templates. The ITK image class is a templated structure over both the pixel type as well as the image dimension. The pixel type is the data type used to represent a pixel. It can be an intrinsic integer or a real number as well as an array-like class to represent vector or color pixels. This design choice pervades all areas of the toolkit. Image filters must be templated over the image type. Also the data structures and objects used with the image classes are templated. These structures include image iterators, points, and indices. The results of this interface design can be seen code Listing 1, taken from the ITK Software Guide (Ibáñez et al., 2005).

**Listing 1:**
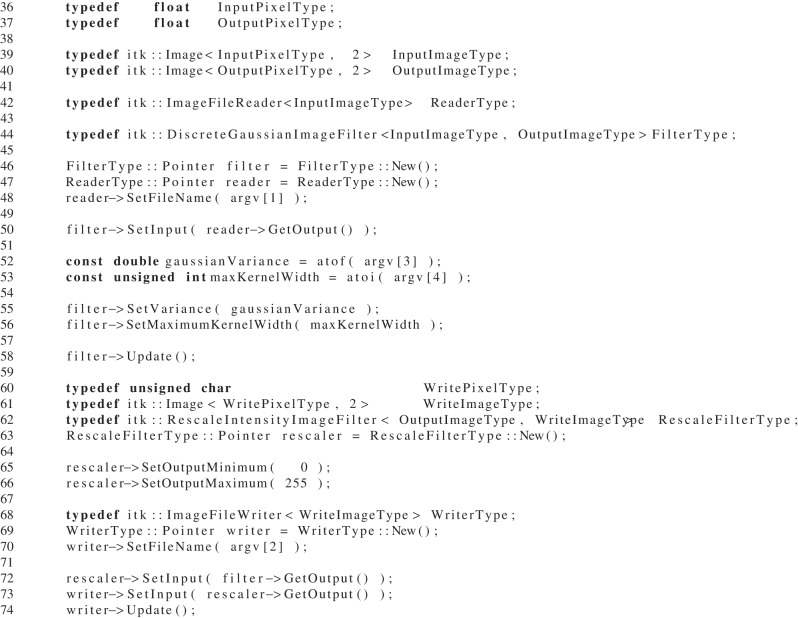
**A typical ITK example with templates, which reads an image, runs a Gaussian convolution then writes it back out to disk**.

Notably missing from the design goals of ITK were usability and ease of accessibility.

### 1.2. WrapITK

The native wrapping of ITK is performed with a project called WrapITK (Lehmann et al., 2006), which was integrated with version 4 into the main source repository. Its goal was to be a direct mapping of ITK's interface for languages such as Python, Java and Tcl. As a direct mapping it attempts to expose all of ITK's functionality including the pipeline, template parameters of data objects and filters along with ITK's use of pointers to objects. WrapITK provides interfaces to many basic ITK objects such the basic array, index vector, point and matrix classes. Additionally interfaces to the core data object types such as images and transforms are provided along with iterators and other utility classes. Most of these types have template parameters for both dimension and value type. The available types in WrapITK make it possible to use most of ITK's filters and many of ITK's modular frameworks, such as registration, with the native ITK class to specify parameters.

WrapITK's implementation was an evolution of the initial ITK wrapping system which used CABLE (Lehmann et al., 2006). There are four components to this system CMake (Martin and Hoffman, 2003), GCC_XML (http://www.gccxml.org), pygccxml, and SWIG (Beazley, 2003). The process is driven by CMake which runs GCC_XML to produce an easily parsable description of the ITK source code, then uses manually written CMake “wrap” files along with Python's pygccxml module to create a SWIG interface file and describe how the ITK templates parameters should be defined. Next SWIG generates the C++ code which instantiates and wraps the ITK for the targeted language.

The result is a powerful exposure of the ITK interface, along with many of ITK's difficulties and problems. Each templated ITK class is instantiated, compiled and wrapped as separate objects. So in the target language the correct template parameters must be provided as part of the class's name to be constructed. While this approach is very much in the style of ITK, many of the targeted languages are scripted and typeless, making this extra verbosity unnatural and cumbersome. This approach also has a very large impact on the size of the WrapITK library and the number of symbols in the library. For every ITK class, with every combination of template parameter, for each method a wrapper method is needed, which introduces multiple symbols in the library that must be loaded into the target language namespace. With most of the pixel types instantiated, the WrapITK library can be over a gigabyte, can contain nearly 3 million symbols, and can take over a minute to load into Python. Also because of the size, the large number of configuration options, and the compile-time and configuration options, precompiled binaries are not available.

### 1.3. Other wrapping

ManageITK was developed to provide wrappers for ITK for the.NET languages such as C#, VB.NET and IronPython (Mueller, 2007). It was based on the WrapITK infrastructure, without the use of GCC_XML or SWIG, instead relying on a manual description of properties of ITK classes, with the addition of some manually written wrapper classes for key data types. Many of the advantages of this interface come from the power of.NET including rapid GUI development, support for multiple languages, and object browsing in an IDE. However, this wrapping approach only works with Microsoft Visual Studio on the Windows operating system, and properties and methods for a new ITK object need to be manually added and updated. Also there is no testing generated for the wrapped interface.

MATLAB (Mathworks, Natick, MA) is a popular commercial platform for research and prototyping bio-medical image processing and other numeric computation algorithms. There are several libraries available to access ITK algorithms inside MATLAB, although they are not providing a direct access to the ITK interface. These include MatITK (Chu and Hamarneh, 2006) and SimITK (Dickinson et al., 2011).

## 2. Design goals

SimpleITK is designed to reduce the burden of usage and expand the ITK user community by simplifying the complexities that are frequently encountered when trying to use ITK. Our goal is to expose the algorithms in ITK in a readily available format. Currently a direct ITK user must be a sophisticated developer with computer science skills to be able to compile and combine ITK algorithms with their own C++ code. While applications (Osirix, Paraview, SNAPITK, 3D Slicer) are built on top of ITK, internally using ITK data structures and algorithms and externally exposing certain filters for image manipulation, users of these applications are not direct users of ITK. We wish to expand the user base, by making the programming interface accessible to non-computer scientists such as domain scientists, biomedical engineers and mathematicians.

Interactive scripting and programming environments such as the Python's SciPy environment (Jones et al., 2001), MATLAB and The R Project for Statistical Computing are popular choices for initial prototyping and experimentations. Scripting languages are easily accessible to more people than C++.

The first complication a new user of ITK encounters is the lack of a binary distribution or compiled software. They must download and compile ITK from the source code, which requires development tools such as a compilers and CMake which may be new to the user. This requirement is a large initial hurdle, and problems are frequently encountered as shown by the many help requests on the ITK community mailing list.

A second common difficulty is dealing with the ITK's advanced C++ templates. ITK uses templates for both an image type's pixel type and the dimension, and the algorithms are templated over the image types. The naive usage of these features require the developer to determine static types used at compile time. The code needed to create a basic program is excessively long and verbose with basic programs requiring numerous C++ type definitions. Handling multiple pixel types or image types can result in even more bulky and complicated code with nested case statements for template function dispatching.

A third problem is complications arising from the ITK pipeline. When filters in a pipeline get executed, the number of times they get executed, and the implicit buffering occurring between filters can all result in performance issues. Common problems include data or meta-data being out of date, excessive memory usage due to filters buffering output, and unnecessary re-execution of filters. In many cases a naively assembled ITK data pipeline can perform worse than sequentially executing filters.

### 2.1. Survey and architectural review board

Kitware conducted a survey of the ITK and related computer vision and medical imaging communities. A variety of mailing lists were queried for users to voluntarily fill out a survey including ITK, VTK, R imaging group, LinkedIn, etc. Feedback was obtained on commonly used tools, applications and requirements for image processing. There were 253 total participants of which 45 participants had never used ITK and 54 were uncomfortable using C++.

The survey produced several important conclusions. First, a pre-compiled, installable package greatly increases the likelihood user uptake. Second, 1 dimensional images are not important with only 4 participants saying it would likely to be used. Third, for each pixel type the group was asked to rate its priority from “Not important” to “Essential.” Every pixel type had a minimum of 25% of the participants saying it was at least a “High Priority.”

When asked questions about preferred programming languages, programming styles, object oriented vs. procedural, features of ITK used or resource requirement, there was a lack of clear consensus. So we concluded that it is important for SimpleITK to be flexible in its usage.

To obtain more specific guidance on the design an Advisory Review Board (ARB) was assembled consisting of 9 members from academia, corporations and government agencies. During the design process pseudo code of interfaces were reviewed. The ARB was another important voice in the design process, giving opinions on best approaches and guidance on prioritizing design philosophies.

### 2.2. Goals

For a successful project there are certain software engineering goals that must be achieved for maximum impact. Firstly, the software should appeal to the greatest audience possible. This goal suggests that SimpleITK must be cross platform and support modern desktop operating systems such as Microsoft Windows, Apple OS X, and GNU Linux. As observed in our survey, a variety of languages should be supported and the framework should easily be extendible to other target languages. Also, to make it easiest for potential users to try the software, there should be binary, downloadable packages available.

Reliability and quality of the software is also of high importance. If a potential user downloads the software, and the first thing they try does not work, they may never invest the time to resolve the issue. To achieve reliability, automatic and manual testing is a must. The coverage of the code needs to be high to build confidence in the quality and reliability of the software.

Given that we want to expand the ITK community beyond those who are comfortable with the current complexities of ITK, certain features should be hidden to enable clearer access to the core algorithms available in ITK. A common complaint about ITK is the difficulties with using the templates for image and filter algorithms. To address this complaint an important objective is to present a template-less abstraction or typeless layer to the native ITK interface that implicitly handles the ITK templated types. We set the larger goal of not exposing any templates in the SimpleITK interface.

Another challenge in our design is to provide a procedural interface to the algorithms, while still providing a object oriented interface for those that prefer it. Therefore the interface design needs to be flexible so that it can support multiple usage styles.

There are many aspects of ITK that we would like to retain. The algorithms still needed to be at the high performance standard that ITK currently has as a compiled C++ library. And we still would like support for the flexible multi-threaded infrastructure when the filtering algorithms are executed. The survey participants placed a high priority on the large number of pixel types available in ITK. Therefore we must support this wide variety of pixel types including color images and vector component images.

The initial mission of ITK included providing a platform for algorithm development, SimpleITK goals focus on providing usable algorithms. So the building block that ITK provides for algorithm development such an image iterator, adaptor, neighborhood algorithms and other utilities and interfaces are not designed to be included in the SimpleITK interface. This limitation should not exclude SimpleITK from being a platform for new algorithm development. For example new methods can be developed that are a composite of other algorithms.

Lastly, while ITK is an biomedical segmentation and registration library, it explicitly does not contain any visualization. That is a user can not view an image with ITK alone. Viewing an image requires an external program or library. However for SimpleITK to be part of an interactive environment convenient visualization of intermediate images is required.

## 3. Implementation

We implemented SimpleITK as an interface on top of ITK, built as a C++ library. This interface is then wrapped for a variety of targeted languages. The SimpleITK interface is functionally complete and fully encapsulates ITK. In other words SimpleITK can be used independently without any direct calls to ITK. This interface was designed from the ground up to be easy to use and intuitive, as well as to take advantages of advanced language features and convenience.

The goals for this project were high, and some of them seemed in conflict. The challenges of presenting a procedural interface for the highly object oriented and templated ITK library while still preserving robust support for multiple image dimensions and pixel types was the essence of the problem in designing SimpleITK. Based on our motivations and goals a number of choices were made.

### 3.1. Decisions

#### 3.1.1. No exposed pipeline

One of the first decisions was that we were not going to expose ITK's demand driven pipeline. We decided to hide the pipeline because errors in using the pipeline are quite common to new users. Without the pipeline, results of operations are immediate so there is no chance that an image is out of date or missing information. Users can simply call the methods needed to set a filter's parameters then execute the filter. The overhead required to connect, manage and update filters is removed from the interface. Also, with filters executing immediately, the object oriented and the procedural interfaces can be closely related.

#### 3.1.2. Use SWIG for wrapping

one of the fundamental goals of simpleITK was to provide language bindings for different languages such as python, java, and c#. Due to the large number of language targets, a single unified tool for all languages was required for maintainability. The simplified wrapper and interface generator (SWIG), is open source and a powerful development tool for wrapping C++ with numerous high-level programming languages. SWIG has support for over 20 target languages. It is capable of parsing basic C++ interfaces and generating glue code to connect the target languages to the interface. The result is generally a direct mapping of a methods from the target language to an associated method in the C++ interface.

WrapITK uses SWIG to interface ITK with other languages. But the complexity of the templated programming used in ITK means that SWIG cannot directly wrap ITK. Instead WrapITK requires additional tools to explicitly specify the interface of ITK in a format that SWIG can understand to wrap certain instantiations of the templated ITK interface. SimpleITK's simplified interface is designed to be directly wrappable by SWIG.

#### 3.1.3. Parameter types

ITK uses a variety of array-like types such as arrays, vectors, indexes, sizes, points and offsets. These types have template parameters such as dimension and value type which makes them dependent on the type of the image, resulting in dozens of array-like types in the native ITK interface. SWIG has built in support for many c++ standard template library (STL) objects such as *std::vector* and *std::list* and provides specialized interfaces for target languages. For example swig can provide an interface to a *std::vector* in python similar to its own list or tuple type along with implicit conversions. This feature greatly improves the interaction in the target language, giving the interface a native feel. Because of the goal to create a template-less layer free from compile-time parameters, simpleITK uses *std::vectors* for array-like parameters for its interface. However, for the targeted scripting language it may appear as native arrays.

#### 3.1.4. Hide smart pointers

Pointers and explicit memory management are elements of C++ that preclude it from being considered a high-level language compared to scripting languages like Python. The smart pointer design pattern is a combination of implicit reference counting for objects along with an interface of a standard pointer so that objects can be automatically deleted when no longer referenced. Thus, they reduce the burden of explicit memory management. While smart pointers are used in ITK, they are not simple enough for SimpleITK as they may introduce a new concept in target languages. Many languages provide a direct object type, not a separate pointer type that refers to the object. The burden of a direct approach in C++ is that implicit deep copies of objects occur during assignments and passing arguments by value. We decided that SimpleITK would provide direct image, filter and transformation objects but not provide the undue burden of implicit copying.

#### 3.1.5. Template-less layer

The template-less or typeless layer concept's goal is to hide ITK's template parameters from the user. A common suggestion was to simply consider all images as 32-bit or 64-bit real numbers. However, this approach can cause unacceptable memory use and performance penalties. Consider starting with an image that is an 8-bit and 4 gigabytes. Converting to a 64-bit type would use eight times the memory now requiring 32 gigabytes of memory for its representation. This monolithic choice changes the nature of working with an image from one that can be done on a laptop to one that requires a large server. Additionally, the users surveyed demanded the flexibility to use a variety of pixel types.

Therefore the template-less layer concept requires that SimpleITK images all have the same external type and interface, unlike ITK. The details of dealing with the different ITK template parameters is hidden so that the user can generically manipulate any image. An image's pixel type and dimension are handled by SimpleITK internally and not directly exposed to the user, unlike ITK which requires the user to explicitly determine them at compile-time. At runtime a PixelIDValue, in the form of an integer and enumerated type is used to represent a pixel type, which along with the image dimension are intrinsic run-time attributes of the SimpleITK image.

#### 3.1.6. Facade interfaces

The facade design pattern is a software design pattern to present the user with an simplified interface to a larger body of code (Gamma et al., 1995). This approach is how SimpleITK encapsulates ITK filters and data objects, where the body of code encapsulated is the set of template parameters for a class. Our facades internally use ITK objects and call ITK methods, so there is very little additional code. The facade code provides a template-less layer to instantiate and dispatch to the correct templated ITK code. Additional code is used to make the objects of the facade behave natively instead of requiring smart pointers and converting between the templated ITK array-like types and STL objects.

#### 3.1.7. Image buffer access

The ability to easily import and export images into SimpleITK is a critical feature. It enables the interfacing between multiple image processing libraries. We chose to allow the direct exposure of an image's buffer via a raw pointer at the C++ interface. This pointer provides efficient direct access, however its use is inherently unsafe. Therefore it is not directly exposed in wrapped languages. Instead our goal is to expose a native array interface to the buffer in the target language, such as numpy for Python.

### 3.2. The design of the simpleITK image

The image class is at the heart of SimpleITK. It is the most commonly used class and has been specially designed to provide a natural and intuitive user interface across multiple target languages. We followed our principles for intuitive interface driven design by writing numerous example code blocks and discussing tradeoffs with the ARB.

From an interface perspective, the handling of the life of an object, i.e., how the object is constructed, copy and destroyed, is a key aspect of developing an intuitive interface. Because we chose not to expose smart pointers, we use a completely different approach from ITK's *New* factory method. Our approach is simple and translates to other languages seamlessly. We simply directly expose the *Image* class's constructors and destructor without the restrictions applied in ITK. The reasons ITK uses the *New* factory method include: adding flexibility to override a class' implementation and enforcing smart pointers use by preventing stack based allocation. Our approach to the SimpleITK interface does not allow our classes to be directly overridden but still allows ITK class overrides to occur internally. As we encourage the direct use of our *Image* class, the smart pointer motivation is moot as well. An *Image* is able to be default constructed, as an image of size zero. An image of any dimension or pixel type is considered to be empty if its size is zero. The common constructor methods take parameters for the size of the image along with the pixel type and support the option for the number of components per pixel. The dimension of the image is determined by the number of components used for size. How *Image*s are copied is closely related to their constructors, but the relationship between the SimpleITK interface and ITK objects needs to be described first.

The SimpleITK *Image* interface presented, utilizes the facade design pattern in conjunction with the private implementation, “pimpl” pattern (Sutter, 2000). It is a facade in that it provides a unified interface for multiple ITK classes and template parameters. The single SimpleITK *Image* class provides an interface to ITK's *Image*, *VectorImage*, and *LabelMap* classes and supports multiple dimensions and pixel types. However, the SimpleITK *Image* class is not polymorphic, i.e., it is not a virtual base class and does not contain virtual methods. Instead it relies on an internal pointer to a private class in the “pimpl” pattern to provide a unified polymorphic interface to ITK's *Image* class. The “pimpl” image has an abstract base class for the unified interface with templated derived classes to implement the specifics. This “pimpl” image fully encapsulate ITK's templates and instantiates all the ITK image classes. A call from the SimpleITK interface to an ITK image is quite efficient and direct despite the complexity of the patterns used. A call to the SimpleITK image's method, calls the same method in the “pimpl” *Image* through the polymorphic interface, which then calls the ITK method. This dispatch method is direct and free from any conditionals. The SimpleITK *Image* only contains a pointer to the “pimpl” image which only contains a smart pointer to ITK *Image*. This implementation provides an efficient interface with low overhead.

Our interface also allows for direct copying and assignment of the *Image* class in an optimized manner. From a user perspective, the availability of optimized copy methods is generally all that needs to be known. However, we have optimized these methods by using a form of lazy evaluation through a copy-on-write (COW) policy. This policy is a key component that allows for an efficient implementation while removing pointers from the interface. Ironically, COW is implemented by using ITK's smart pointers. When a copy of a SimpleITK *Image* occurs, a new ITK smart pointer to the image is created and stored. This new smart pointer increases the reference count contained of the ITK *Image*. When there is a write request to the SimpleITK *Image*, the reference count is checked; if it is not 1, then a deep copy is performed of the bulk image data. This approach enables the SimpleITK *Image* to be passed by value without the overhead of implicitly copying the bulk image data.

Our user survey and ARB discussions were inconclusive in determining a limited set of important pixel types to implement. As adding more image or pixel types does not increase the number of methods in the SimpleITK interface, we do not limit the types enabled. Currently we have up to 26 different pixel types available including scalars, multi-component vectors, complex as well as label maps implemented with the run length encoded *LabelMap* classes. Fully implementing these type does not have an adverse effect on usability or run-time. It does however increase the demands on the system requirements for compiling the library. As the goals for SimpleITK focus more on usability of the interface then ease of building, it was determined to be a reasonable tradeoff. Additionally, we settled for only supporting 2 and 3 dimensional images. Interest by the users in higher dimensional images was limited.

The *Image* interface was also customized for C++, Python, R, Java and C# to provide additional syntactic enhancements to make language integration as easy as possible. These enhancements include features such as operator overloading, advance subscripting, and utilizing weak typing of return values in many scripting languages.

The resulting image class provides a simple and easy to use interface that fully encapsulates the complexities of ITK. Among the things that are hidden include templates for both the image dimension and pixel type as well as multiple image classes. Details such as smart-pointers, memory management and copy semantics are also removed and replace with a straightforward C++ interface. This standard interface can be directly wrapped by SWIG, thus providing a conventional interface for different target languages.

### 3.3. The design of the simpleITK filters

Among the objectives which drove the filter interface design of SimpleITK included removing the dependency on the image type at compile-time, the removal of ITK's data driven pipeline as well as providing a flexible interface which has an object oriented and procedural interface. With the template-less image class designed and the decision to use STL's *std::vector* as parameters whose length is dependent on the image dimension, the interface was rather straight forward. Determining which types to use for filter method parameters was a difficulty when it was the same type as the image's pixel type. Generically using the double type resolved this issue. The double is a superset of all scalar pixel types except 64-bit integers, and for vector pixel types the scalar value is replicated for all components. Listing 2 can be used as a reference.

**Listing 2:**
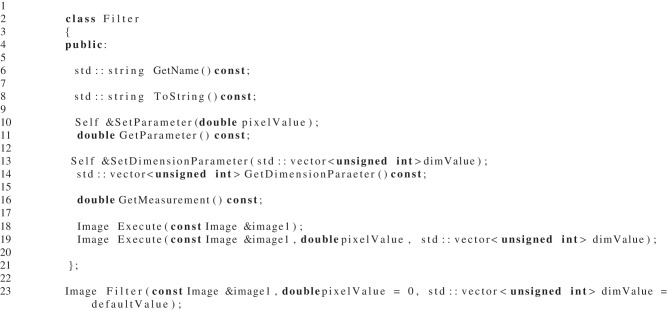
**A prototypical example of a SimpleITK filter interface**.

In ITK, the pipeline needs to be explicitly updated before results can be examined. This update initiates the examination of the dependencies in the pipeline to determine the necessary filters to execute. After the Update method of a filter is called the results in the output are valid and can now be examined. Without the pipeline, a filter is executed immediately, so the method name Execute was chosen to illustrate the difference. Filters which have secondary results, such as a number of iterations or a measurement of error are problematic for the procedural interface as functions naturally only return a single value. The solution was to add “measurements” only to the filter classes. An alternative approach, to make these available through the procedural interface was have them as arguments to the procedural methods. However doing so would result in too many arguments, and passing by reference or pointer is not portable across differing languages.

Defining the behavior of a filter is more complicated than specifying the desired interface for the template-less filter. For each filter decisions must be made about the input types supported as well as the image type produced. The goal is to have filters support as many image types as possible. Even when the ITK filter does not directly support vector images, the SimpleITK infrastructure can execute the ITK filter on each component independently. For determining the output image type there are a couple general policies that occur. The first and most common is defining the output image type the same as the input. This is sensible for many filters including mathematical morphology, image grid manipulations, and label image manipulations. For other filters having a fixed output type is sensible. For example filters which output a mask such as thresholding, always output a unsigned 8-bit integer, while distance field filters produce 32-bit floating point numbers. Consideration must include limiting the number of instantiated filters of one type to a reasonable number, to prevent excessive compilation and library size from excessive permutation, so in general there is no selection of the output type.

Those filters which take two image as input required special consideration for how to handle the multiple types. In ITK, filters that are binary functors such as the addition and subtraction operators, have 3 template parameters corresponding the two inputs and the single output. This approach makes ITK very flexible by allowing conversion to occur simultaneously with the operation. Languages have different type promotions schemes, and certain overflow and conversions are explicitly undefined in C++. Therefore we decided that most of the binary filters require the inputs to both be of the same type as well as the resulting output. So we do not do any implicit conversion or promotion, thereby requiring the user to explicitly cast the inputs to be the same type. SimpleITK does have the ability for filters to accept multiple images of differing types and to use the correct ITK filter templates based on these input types. These filters are called dual image filters in SimpleITK. They have been used sparingly due to size constraints when the computation validity of multiple types is not in question. The types of filters where this feature is used include filters with masking and overlays. The group of filters which allow the user to select the output type such as casting, camping, and resample also use the dual dispatch infrastructure.

By choosing to remove ITK's pipeline architecture from SimpleITK there are numerous simplifications that we have taken advantage in our design. Among the ITK features SimpleITK omits are the advanced details of memory management occurring in the pipeline such as releasing data, and running filters in place. They are not relevant, since SimpleITK has an immediate execution model that lacks pipeline buffering. ITK's interface for the *ProcessObject*, which is the basis for all filters, consists of over 40 public methods for manipulating the pipeline and managing the inputs and outputs. All these methods are irrelevant for SimpleITK's *Execute* method approach. Omitting them simplifies the interface for each filter class. Among the features still relevant is the monitoring of progress and events; these features are scheduled for the next release of SimpleITK, version 0.8.

### 3.4. Implementation details

The design of the interface was not done in isolation from the implementation. Extreme software development methodologies were use to continuously test and regularly integrate features into a stable version. The goals and the design of the interface presented immense software engineering problems that had not been addressed before with ITK. Initially there were doubts that maintainable code and infrastructure could be developed to create a template-less layer for a significant part of ITK's filters without unmaintainable, manually created code. Our resulting implementation uses advanced C++ template meta-programming to perform compile-time execution of code generation, as well as traditional code generation based on the description of filters. The details in this section are completely hidden from a user of SimpleITK, and are relevant to those examining the internals of SimpleITK or those who wish to learn the novel techniques used for similar projects.

#### 3.4.1. Overview

**Figure 1:**
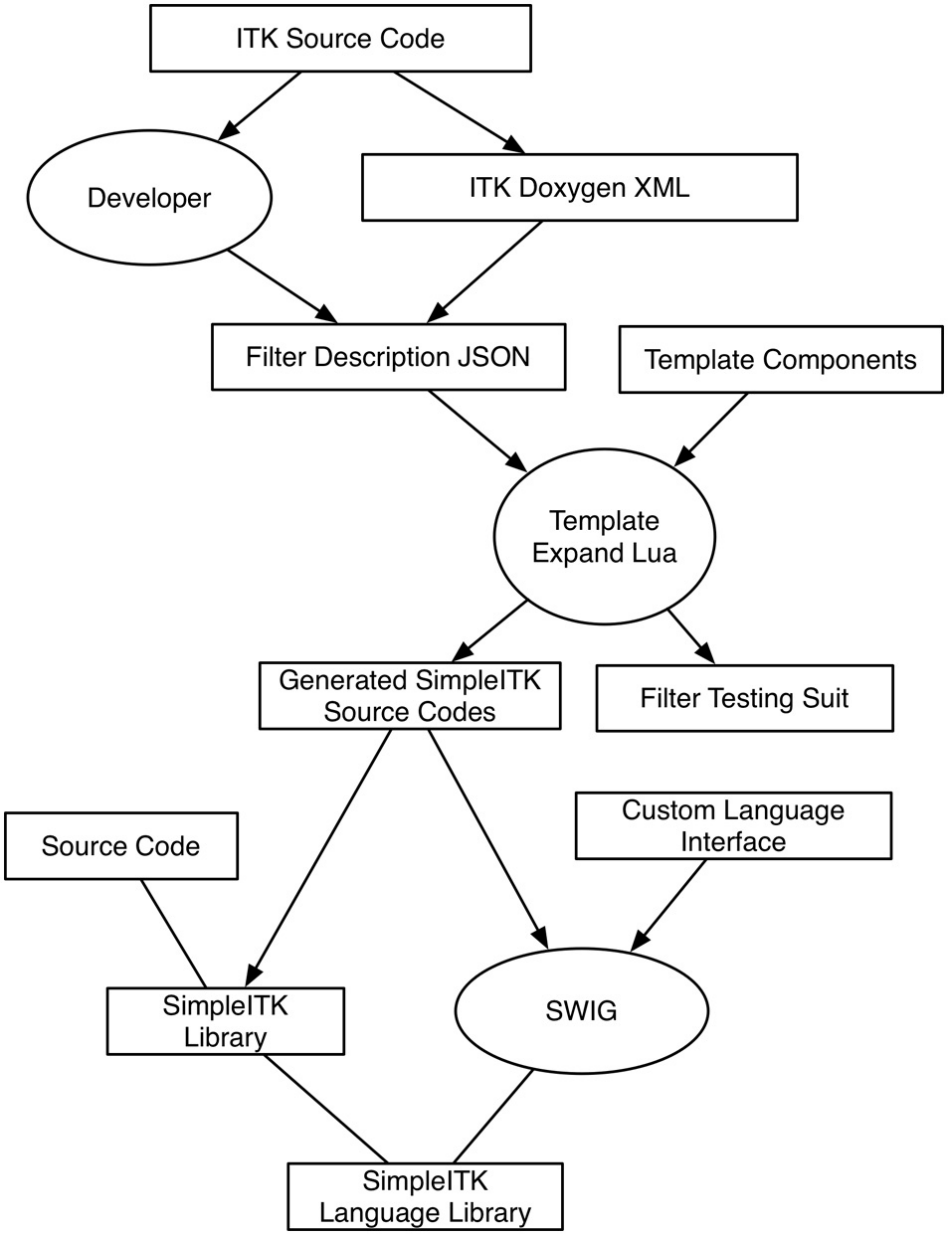
**Data flow diagram of SimpleITK’s build process**. Boxes represent data, while ovals are important processes.

SimpleITK is implemented with more than just static code. It is a complex sequence of code generation and compilation to produce the C++ interface, followed by wrapping with SWIG for the target languages (see Figure 1). The manually written common code of SimpleITK consists of the meta-programing infrastructure for the template-less layer and the core data structures such as the image and the transform classes. Also, a few of the filters and the reading, writing and image display interfaces are written by hand. As of SimpleITK 0.7.1, the SimpleITK C++ interfaces for 245 image filters are automatically generated from manually written descriptions.

#### 3.4.2. Code generation

After experimenting and iteratively improving manually written filter interfaces, it was immediately apparent that a solely manually written interface would take excessive time to develop, lead to an inconsistent interface, and be unmaintainable. Due to the requirement of maintaining a consistent interface among a large number of filters, automatic source code generation became the natural choice. Code generation allows the developers to focus on writing the generator and the description at a higher level than the immediate interface.

Each generated filter in SimpleITK is described by a JavaScript Object Notation (JSON) file. The JSON file structure is a lightweight data format popularized by JavaScript programming for the web, which is easy for both humans and computer software to read and write. The JSON description contains the essence of an ITK filter. A filter description contains expected named fields, such as *name*, *template_type*, *number_of_inputs*, *pixel_types*, and *output_pixel_type*, with values, as well as more generic fields which can contain C++ code to be inserted into the template. The *output_pixel_type* is frequently more that just a static value by containing meta-programing to transform the input type to the output. Additionally, there is the *member* sections which contains the description for each filter parameter so that it can be stored in the SimpleITK facade and translated into the typed ITK filter. There is also an optional section to describe *measurements*, and how to update the member variable from the results of the ITK filter.

For normal image filters previously mentioned portion of the JSON description can generally be written in just a few moments from a similar example. The time consuming part in writing a JSON description is the *tests* section. This section contains input image file names, the values of parameters, and either a hash of the expected output or a baseline image to compare a test against. By including this section we are able to generate tests for each language to verify usability and functionality of the filter with different image types. Automatic testing is critical to developing a robust interface that is proven and reliable.

The generator of the source code is a text based program, which processes a set of text inputs and produces source code. Lua is a powerful, portable, fast, and lightweight scripting languages (Ierusalimschy et al., 2006). We chose it as the language for the source code generator script because its small size makes it easily included as part of the SimpleITK source and compiled during the build process. The generator script is derived from the Lua “Text Template Expand” example (http://lua-users.org/wiki/TextTemplate) combined with an publicly available JSON parser. This system uses template files which contain and inlined Lua expressions all of which are evaluated into strings. The scope of the Lua expressions include variables from the current scope in the JSON description file. There are template files for filters of types such as *ImageSources*, *BinaryFunctorFilters*, and *DualImageFilters*.

#### 3.4.3. Template meta-program and typelists

The crux of the problem for the template-less layer is the ability to map the runtime pixel type information, *PixelIDValue*, available from SimpleITK's *Image* to the compile-time template parameters in ITK and vice versa. Template meta-programming in C++ is the use of templates to perform compile-time execution to generate code. SimpleITK uses a concept called “Typelists” to operate on list of types, *PixelID*s, which represent the types of pixels. Specifically these lists have compile-time operations to append, merge, intersect, search, traverse and perform permutations of selections from two lists (Alexandrescu, 2001). The runtime ID of a pixel type, *PixelIDValue*, is the index in the global *InstantiatedPixelIDTypeList* type, which is a *Typelist* of all the available pixel types. There are a variety of groups of *PixelID*s declared as *Typelist*s such as integer, real and vector. The JSON description of a filter describes the valid input pixel types by performing meta-programming operations on these *Typelist*s.

The compile-time mapping of the run-time *PixelIDValue* to an instantiated function with the image as a template parameter is more problematic. The compile-time mapping of the *PixelID* to the scalar *PixelIDValue* can and must be done on all instantiated types. On the other hand the templated functions can only be instantiated with the parameters that are specified via *Typelist*s. Otherwise the code may not be valid.

The mapping of the run-time *PixelIDValue* to an instantiated member function is done with an abstraction called a *MemberFunctionFactory*. This factory has a method which maps a *PixelIDValue* and image dimension to a member function pointer of an instantiated member function over the associated ITK image types. These functions are registered into the factory by passing a *PixelID Typelist* and the dimension. Additionally the factory has a templated object which knows how to get the address of the member function, enabling the compile-time traversal of the *Typelist* to instantiate the member function. With the member functions stored in the factory, they are indexable by the *PixelIDValue* at run-time. This *MemberFunctionFactory* is the heart of the dispatch system used to implement SimpleITK's templateless layer and is an important software engineering innovation for dispatching run-time mappings to ITK types.

#### 3.4.4. Automated documentation

Without documentation to accompany filters and algorithms, the interface of SimpleITK would be unusable. Duplicating the effort to describe each algorithm and parameters with what has already been done in ITK would be a foolish task. Doxygen is the de facto standard tool for generating reference manuals of a programming interface from annotated source code (http://www.doxygen.org). It is used as the source code annotation throughout ITK and SimpleITK. For all the manually written SimpleITK infrastructure and facade interfaces the annotations are written in the source code. Automated utilities have been developed to insert documentation strings from ITK into the JSON filter description files. These documentation fields are then incorporated into the generated source code as well as used for wrapped target language's online documentation. Currently the ITK documentation sometimes contains C++ implementation details or other features only available in native ITK. In the future ITK's documentation may be further annotated to note the relevance of different sections to allow better integration with SimpleITK and other external projects.

## 4. Examples

### 4.1. Multi-modal ventricle segmentation

Segmentation of the human brain lateral ventricles is a common task in the study of brain anatomy and its diseases. Changes in the volume of cerebrospinal fluid and the shape of the cerebral ventricles are associated with a number of diseases including hydrocephalus (Brandt et al., 1994) and schizophrenia (Staal et al., 2000).

In this example we use SimpleITK's *VectorConfidence Connected* filter (Ibáñez et al., 2005) to segment an MRI data set from the Neuroimage Analysis Center's Multi-modality MRI-based Atlas of the Brain (Halle et al., 2013). This filter begins with user-selected seed points then iteratively grows a region based on the similarity of the local vector image statistics. To create a vector image we combine the MRI T1 and T2 images into a single vector image.

We developed this script interactively in an IPython Notebook (Pérez and Granger, 2007) session. We implemented custom display functions called *showimg* and *showimg3d*, which allow for the easy tiled display of multiple slices. For display we export the tiled slices to *numpy* and utilize *matplotlib* for inline display in the IPython notebook.

**Figure 2:**
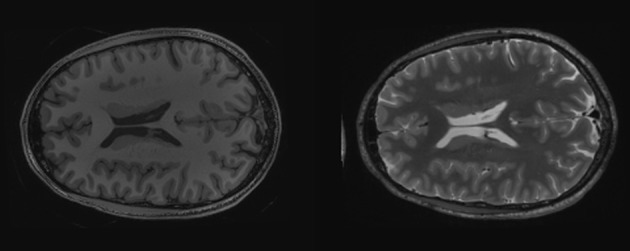
**Axial cross sections of T1 (left) and T2 (right) MRI of a brain**.

First we read the T1 and T2 brain images and create unsigned char version for display (see Listing 3: lines 5–10). Taking advantage of SimpleITK's Python array slicing, extracting axial slices from the MRI volumes is simple (see Figure 2 and Listing 3: lines 13–14). Then from the original T1 and T2 volumes we create a vector volume using SimpleITK's Compose function (see Listing 3: line 18).

**Listing 3:**
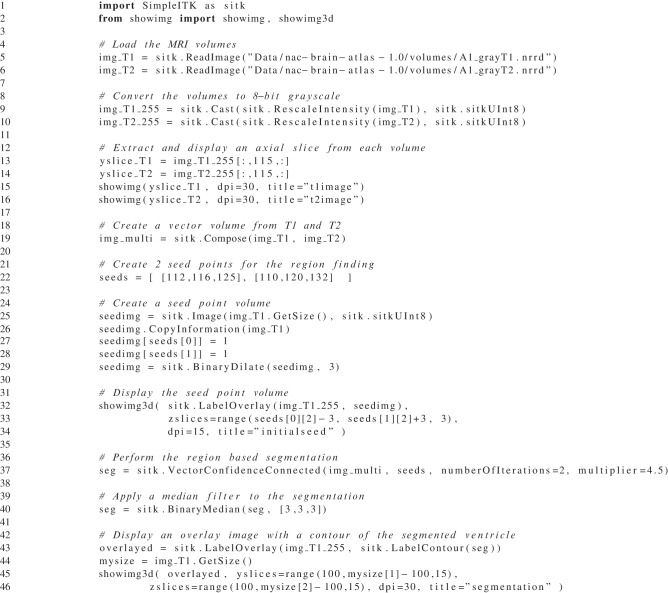
**A Python script using SimpleITK for the multi-modal segmentation of the lateral ventricles**.

**Figure 3:**
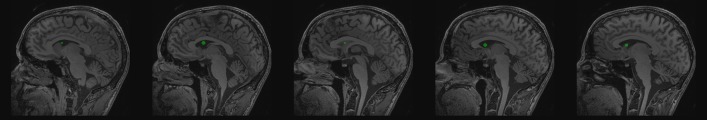
**Segmentation seeds in green on coronal cross sections of T1 volume**.

For initializing the segmentation of the cerebral ventricles we start with 2 seed points, one each side (see Listing 3: line 21). For visualization purposes, we create a seed volume, based on the dimensions of the T1 volume. Setting the seed voxels to 1 is simple with SimpleITK's array indexing (see Listing 3: line 25–29, and Figure 3).

**Figure 4:**
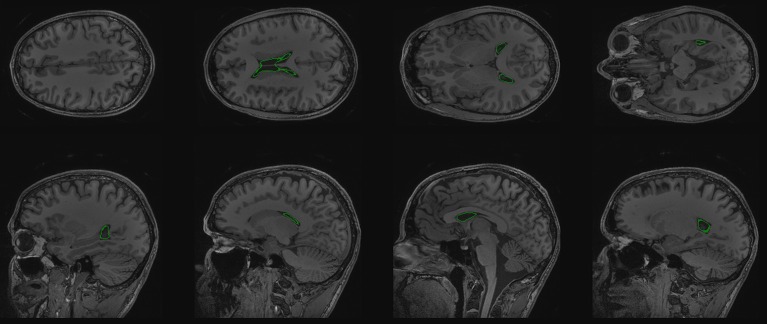
**Axial and coronal cross sections with the resulting ventricle segmentation shown as a green contour**.

To display our segmentation we can create a contour of it and overlay that on the T1 volume (see Figure 4 and Listing 3: line 43–47).

**Figure 5:**
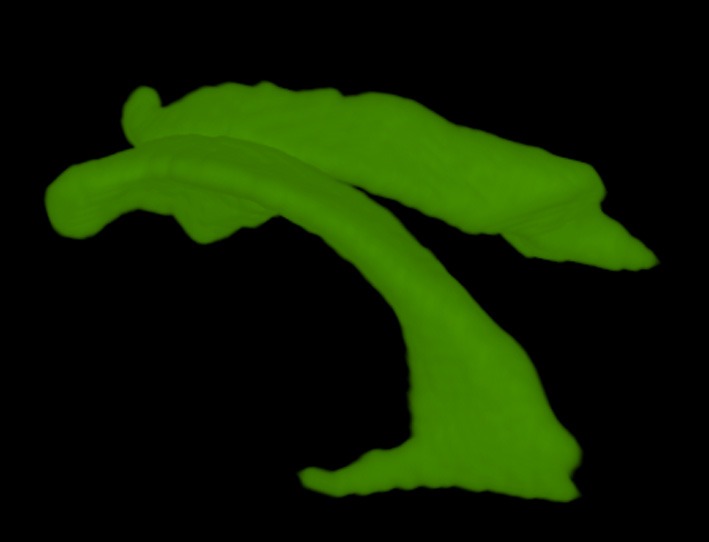
**Volume rendering of segmented ventricle**.

Finally using a volume renderer we can visualize the lateral ventricles segmentation in 3d (see Figure 5).

### 4.2. Microscopy segmentation

Many modalities of microscopy acquire images at high resolution over large areas and can result in very large data sets. The Connectome Project at Harvard's Center for Brain Science is one example (Seyedhosseini et al., 2011).

To illustrate the computation efficiency and the effectiveness of using SimpleITK on larger real world problems, this example demonstrates a segmentation of an electron microscopy dataset. Ion-abrasion scanning electron microscopy (IA-SEM), is an image acquisition technique from the combination of traditional scanning electron microscopy acquired on a sample block with a focused ion-beam used to mill the block with nano-meter resolution. The method has been used for whole cell imaging (Murphy et al., 2011).

**Figure 6:**
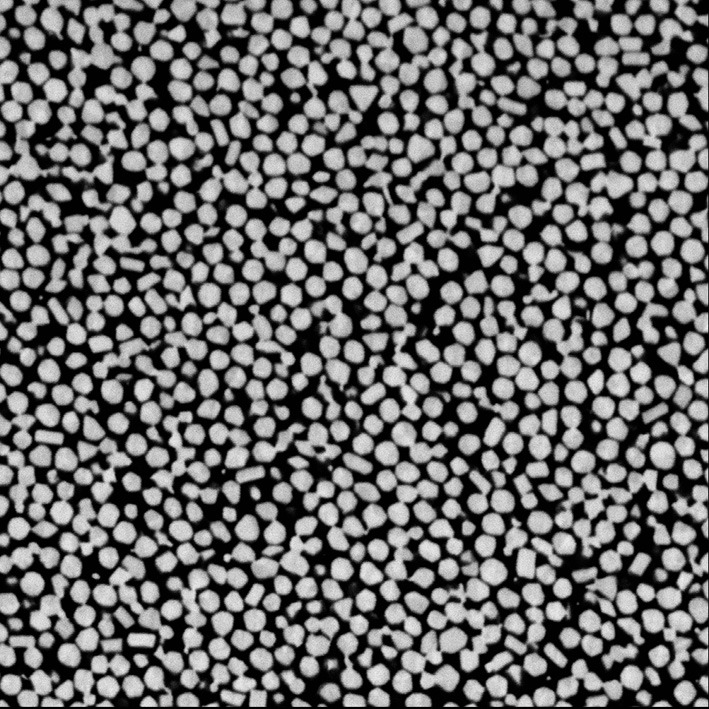
**Slice from the gold bead volume with 5nm pixel resolution**. The dense 100nm gold beads are bright while the embedded resin is dark.

The dataset chosen is a volume of 100 nm gold beads used to analyze the procedures and methods for data acquisition (see Figure 6). It was collected by the Biophysics Section and Electron Microscopy Core, Laboratory of Cell Biology at the National Cancer Institute and is publicly available on a MIDAS data server at the NLM (http://placid.nlm.nih.gov). The dataset was acquired at 5 nm square voxels with a volume size of 1003 × 1003 × 296 pixels resulting in a 284 megabyte volume.

The goal of this segmentation is to identify each distinct gold bead by creating a unique label for each gold bead. The segmentation occurs primarily in two steps: first identifying the individual beads (see Listing 4: lines 9–16) and second growing the seeds (see Listing 4: lines 26–27). This sequence of processing was developed in an interactive python session making use of SimpleITK's *Show* method to visualize intermediate results. It was then converted into a single script to assist with batch processing and parameter exploration.

**Listing 4:**
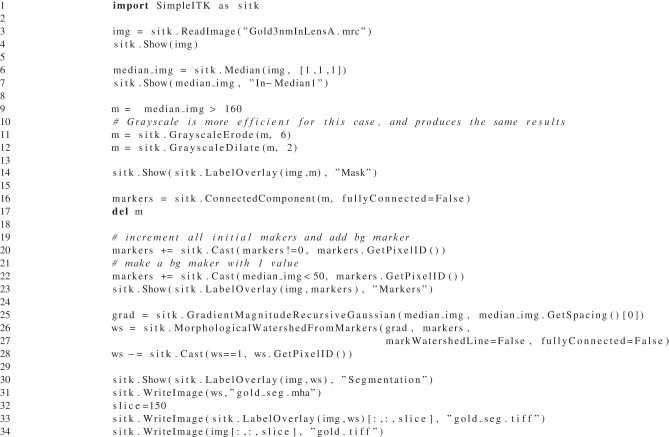
**Example Python script using SimpleITK to segment gold beads with the watersheds algorithm**.

The method first uses thresholding to identify the high intensity gold, then uses mathematical morphology to separate the beads of at least 13 pixels (65 nm) in diameter. Next, each isolated part is given a unique identifier using the connected components algorithm. The watershed from markers algorithm available in ITK (Beare and Lehmann, 2006), is a multi-label greedy region growing algorithm subject to the penalty of the feature image. The individuated gold components are combined with an additional marker for the background. This marker image is used in conjunction with an edge image for the watershed algorithm. The SimpleITK code is easy to follow, brief and executes 16 ITK filter to perform its task. Many of those filters are invoked through overloaded operators for comparison and arithmetic.

**Figure 7:**
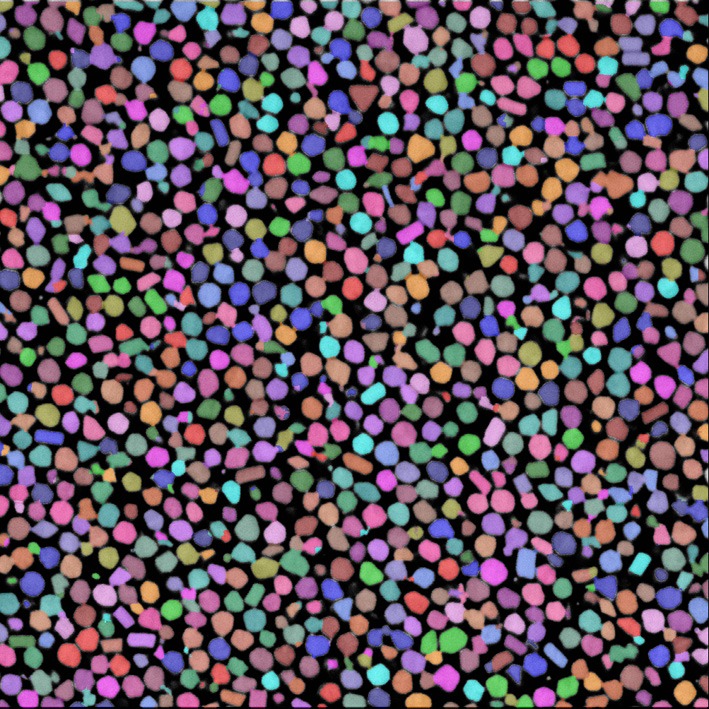
**Gold bead segmentation results on the same slice as Figure 6**. Each identified bead has a unique color transparently overlaid the original image.

**Figure 8:**
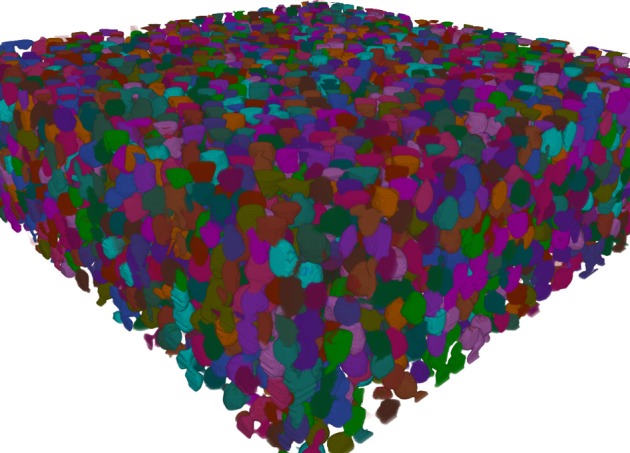
**Volume rendering of colorized labeled gold bead segmentation**.

The resulting segmentation consists of over 9000 segmented gold beads (see Figures 7 and 8). Segmentation is just one of many steps in the process of quantitative analysis. SimpleITK provides facilities for basic statistics in the *LabelImageStatisticsImageFilter*. However, for this study custom shape analysis was implemented in C++ within ITK's *LabelMap* framework, illustrating the complementary nature of SimpleITK's and ITK's interfaces. When implementing new algorithms ITK can be the best tool to use, while interactively exploring existing algorithms for a solution may be better suited to SimpleITK.

### 4.3. Simplefilters in 3D slicer

The SimpleITK infrastructure can also be used as a way to integrate ITK into applications. Many image processing applications are designed in a modular fashions so that modules or extensions can be created to add new functionality. A frequent objective is to integrate ITK's important algorithms into an end user application with a graphical user interface (GUI). When trying to directly integrate ITK with applications, there are common problems encountered that SimpleITK can resolve. Interfacing an application's data types with the templated ITK types requires dealing with template parameter instantiation and run-time dispatching. Also, for each filter or algorithm important parameters and default values need to be determined. SimpleITK can help with these and other difficulties to enable rapid integration of a large number of ITK filters into a application.

3D Slicer is in modular image computing platform used for quantitative analysis in medical image computing and clinical research (Fedorov et al., 2012). It is a free and open source application with support for numerous biomedical imaging formats and functionality such as automatic interactive segmentation, registration, and visualization. It is built upon a variety of open source software to provide core functionality such as visualization, GUI support, and script. It has a modular architecture to support custom plug-ins. Despite 3D Slicer including ITK in its C++ backend, the burden of creating a module for a large number of ITK filters was too great, so many of ITK's basic image processing algorithms were not available to end users.

The SimpleFilters module in 3D Slicer was created to provide a basic GUI to the image filters available in ITK through SimpleITK. During the course of a single week at a National Alliance of Medical Image and Computing (NAMIC) project week, with the support of various member of the developer community, we created an initial implementation of the module encorporating over 200 SimpleITK image filters with GUIs for their parameters.

The module was written in Python to take advantage of the ability to generate a string and evaluate that string at runtime. By using this run-time capability with a few hundred lines of code, we were able to implement code to generate a GUI for the parameters and to execute each filter. This module was created without any filter specific code or compile-time code generation, but by reading SimpleITK's JSON descriptions files to obtain the parameters. The calls to configure filters for execution were constructed at runtime from strings. Also the documentation strings in the JSON are used for mouse quick tips and hover overs. The module uses SimpleITK and its template-less layer for runtime type dispatching, so all image types are supported without additional case or switch statements. The SimpleFilters module has been incorporated into 3D Slicer and is part of the binary distribution with version 4.3.

The JSON filter descriptions in SimpleITK contain the essence of ITK. They provide a standard to describe the parameters for algorithms, along with their default values and documentation. In addition to the interface and language bindings that SimpleITK provides, these descriptions are another valuable asset when integrating ITK into applications. They can dramatically reduce the implementation time and increase the number of algorithms that can be made available.

## 5. Discussion

The SimpleITK interface with wrapping for additional languages is a new and easy way to use ITK's image processing algorithms that will make these algorithms available to a wider audience of domain scientists. A number of features of ITK are hidden and the bulk of the interface has been simplified. Currently approximately 250 algorithms are available through SimpleITK for interactive exploration in a number of scripting and prototyping environments.

SimpleITK has already gone through several releases during development. The most current release available is 0.7.0rc1. As of this writing there have been over 7000 binary downloads of SimpleITK on SourceForge. This number does not include those who have built SimpleITK from the source code repository or are using it from an integrated application. The SimpleITK official website (http://www.simpleitk.org) contains more information about how to get started using SimpleITK.

### 5.1. Future work

While the foundations of ITK have been integrated into the SimpleITK interface for classes such as images, transforms and interpolators and the infrastructure for source code generation for filters has been implemented, much of the work for ITK's registration framework is yet to be done. Some ITK registration methods are ITK filters and can easily be integrated as part of the current framework. The modular registration framework in ITK version 3 and the latest version, v4, present new problems which require novel design solutions.

Additionally, there are features in ITK that still need to be incorporated in to the SimpleITK interface such as progress reporting and call-back methods. While SimpleITK currently contains over 250 image filters, we are always looking for feedback from our user community for what additional ITK filters should be priorities in future work. Many of the unincorporated ITK filters are not commonly used. So when the SimpleITK interface is tested, bugs in ITK may arise or other inconsistencies may be discovered in ITK's interface. These issues must be addressed and fixed in ITK and slow the development of SimpleITK.

ITK is a very large toolkit consisting of millions of lines of code. The SimpleITK interface is highly customized and not designed to expose all of ITK. The design goals of SimpleITK have been for a robust, reliable, and elegant interface to ITK. To that end we have focused on the template-less layer to the image class and image filters. There are many additional data structures that could be added to the interface including histograms and quad-edge meshes. However, we wish the presented interface to be the best possible. To that end some portions of ITK may be avoided to maintain a high standard until there is broad ITK community support.

The number of the languages which SimpleITK is wrapped for is large and includes Python, Java, C#, Ruby, Lua, Tcl and R. Developing and maintaining unique features and “syntactic sugar” such as advanced subscripting and native buffer interface for all languages is too large of a task. We seek the on going support of the ITK community to support these features and languages. Specifically, in the near future we hope to add native Java array integrations with SimpleITK image buffers and improve SWIG's wrapping for the R language to handle enumeration types correctly.

### 5.2. Conclusion

SimpleITK is a new user-friendly software interface to the image processing algorithms available in ITK. Through the use of innovative software engineering combined with disciplined testing practices, SimpleITK makes a large portfolio of reliable open source imaging algorithms available to a broad range of scientific communities. The end goal of this work is not the SimpleITK software itself. Rather the goal is to aid research over a wide range of scientific fields by improving accessibility and reproducibility for state of the art image analysis algorithms.

### Conflict of interest statement

The authors declare that the research was conducted in the absence of any commercial or financial relationships that could be construed as a potential conflict of interest.
